# Schottky barrier height engineering on MoS_2_ field-effect transistors using a polymer surface modifier on a contact electrode

**DOI:** 10.1186/s11671-023-03855-z

**Published:** 2023-05-31

**Authors:** Dongwon Choi, Jeehoon Jeon, Tae-Eon Park, Byeong-Kwon Ju, Ki-Young Lee

**Affiliations:** 1 Center for Spintronics, Korea Institute of Science and Technology, Seoul, 02792 South Korea; 2 Department of Electrical Engineering, Korea University, Seoul, 02841 South Korea

**Keywords:** MoS_2_ FET, Polyethylenimine, Schottky barrier height, Thermionic emission, Photoelectric effect

## Abstract

Two-dimensional (2D) materials are highly sought after for their superior semiconducting properties, making them promising candidates for next-generation electronic and optoelectronic devices. Transition-metal dichalcogenides (TMDCs), such as molybdenum disulfide (MoS_2_) and tungsten diselenide (WSe_2_), are promising alternative 2D materials. However, the devices based on these materials experience performance deterioration due to the formation of a Schottky barrier between metal contacts and semiconducting TMDCs. Here, we performed experiments to reduce the Schottky barrier height of MoS_2_ field-effect transistors (FETs) by lowering the work function (*Ф*_m_ = *E*_vacuum_ − *E*_F,metal_) of the contact metal. We chose polyethylenimine (PEI), a polymer containing simple aliphatic amine groups (–NH_2_), as a surface modifier of the Au (*Ф*_Au_ = 5.10 eV) contact metal. PEI is a well-known surface modifier that lowers the work function of various conductors such as metals and conducting polymers. Such surface modifiers have thus far been utilized in organic-based devices, including organic light-emitting diodes, organic solar cells, and organic thin-film transistors. In this study, we used the simple PEI coating to tune the work function of the contact electrodes of MoS_2_ FETs. The proposed method is rapid, easy to implement under ambient conditions, and effectively reduces the Schottky barrier height. We expect this simple and effective method to be widely used in large-area electronics and optoelectronics due to its numerous advantages.

**Supplementary Information:**

The online version contains supplementary material available at 10.1186/s11671-023-03855-z.

## Introduction

Recently, two-dimensional (2D) layered materials have attracted significant interest because of their unique electrical, optical, mechanical, and chemical properties [1–5]. Since the first isolation of graphene, which was achieved by mechanical exfoliation, there has been numerous advancements in the research field of 2D materials [6, 7]. The excellent properties of graphene have spurred interest in other 2D materials [8–10]. Molybdenum disulfide (MoS_2_) is a typical layered transition-metal dichalcogenide (TMDC) with the general formula *MX*_2_, where *M* is a transition metal (Mo, W, Zr, Hf, or Nb) and *X* is a chalcogen (S, Se, or Te) [11–14]. Similar to graphene, which exhibits weak van der Waals forces [15, 16], single-layer MoS_2_ has a structure in which S-Mo-S layers are stacked on top of each other [17, 18]. Two-dimensional MoS_2_ has been utilized in the development of various devices such as field-effect transistors (FETs), electrically driven optical devices, electrochemical devices, and solar cells [1, 13, 19–22]. In addition, it is emerging as a promising candidate for use in future nanoelectronic and spintronic applications.

The performance of a device based on MoS_2_ is significantly influenced by the metals chosen to form electrical contacts. The behavior of *n* (*p*)-type MoS_2_ transistors is regulated by electron (hole) injection from the metal contacts, and comprehending the intrinsic electrical transport properties of the MoS_2_ channel necessitates the use of ohmic contacts. It is challenging to demonstrate *p*-type MoS_2_ transistors because high work function metals (e.g., Au, Ni, and Pt) exhibit *n*-type behavior in MoS_2_ transistors [23].

At the junction between a metal and a semiconductor, when the work function (*Ф*_m_) of the metal is higher than the electron affinity (χ) of the semiconductor, a potential energy barrier for electrons is produced, known as the Schottky barrier [24, 25]. The height of this barrier can be calculated by substracting the work function of the metal from the electron affinity of the semiconductor relative to the vacuum level. To construct the energy band diagram accurately, it is essential to align the energy bands of both the metal and semiconductor by using the same vacuum level as the reference. At the state of thermal equilibrium, the Fermi energies of the metal and semiconductor must be equal when they come into contact. The height of the barrier can be defined as the potential difference between the Fermi energy of the metal and the band edge where the majority carriers reside. The barrier height for *n*-type semiconductors is calculated using the equation *Ф*_B_ = *Ф*_m_ − χ. The estimation of the *Ф*_B_ value is releant on the specific combination of metal-semiconductor. The formation of the Schottky barrier is a complex problem because metal-semiconductor contacts are often dependent on the difference between the semiconductor electron affinity and the metal work function.

Achieving a low contact resistance by reducing the Schottky barrier height between MoS_2_ and the metal contacts, which is strongly related to carrier mobility, is critical to the development of future 2D electronics and optoelectronic devices but remains challenging. Extensive efforts have been devoted to reducing the Schottky barrier height and improving the contact properties of MoS_2_, including the use of low work function contact metals [23, 26], inserting atomically thick hexagonal boron nitride (h-BN) as a tunneling layer [27], molecular/chemical doping of MoS_2_ [28–30], and inserting a graphene film between the electrode and MoS_2_ [31]. Das et al. have reported on their investigation of the contact resistance between MoS_2_ and four different metal contacts: Sc, Ti, Ni, and Pt. Their findings indicated that using Sc as a contact electrode resulted in the lowest contact resistance owing to its low work function metal properties [23]. However, the presence of a van der Waals gap at the MoS_2_-metal interface can cause high contact resistance as a result of tunneling [32]. Moreover, the use of doping strategies may cause MoS_2_ to be unstable in ambient air and lead to complete channel doping, resulting in a decreased on/off current ratio due to significant leakage current [28–30].

In a previous study, Zhou et al. used a surface modifier to prepare a low work function electrode suitable for diverse electronic devices [33]. According to their study, the inherent dipole moments of neutral amine groups within an insulating polymer layer, along with the charge transfer properties that arise from their interaction with the conductor surface, can significantly lower the work function of a wide variety of conductors. They tested low work function electrodes with various organic solar cell (OSC) geometries. The OSCs were fabricated with a surface modifier as the bottom electrode to demonstrate electron selectivity. Solar cells with surface modifier-coated indium tin oxide (ITO) electrode yielded a power conversion efficiency (PCE) of 5.9% [open-circuit voltage (V_OC_) = 0.81 V, short-circuit current density (J_SC_) = 11 mA/cm^2^, fill factor (FF) = 0.66] [33]. Such a large FF value provides indirect evidence of the good electron selectivity of the surface-modified ITO electrode. In this study, we aimed to reduce the Schottky barrier height by applying a method described in reference [33], which involves the physisorption of an ultrathin layer of a polymer containing simple aliphatic amine groups onto the surface of the contact electrodes, with the expectation of reducing the work function of the contact metal. The proposed method can be easily executed under ambient conditions using dilute solutions prepared with environmentally benign solvents such as water or methoxyethanol. Due to its cost-effectiveness and facile processability, this approach is amenable to roll-to-roll large-area mass production techniques, rendering it suitable for the fabrication of organic or printed electronic devices.

## Experimental

### Fabrication of MoS_2_ FETs

MoS_2_ flakes were mechanically exfoliated from bulk crystals using the “Scotch tape” method and were then transferred to a heavily *p*-doped Si substrate with a 285 nm-thick SiO_2_, which functioned as a back gate and gate dielectric, respectively. After observing the MoS_2_ flakes by optical microscopy, we characterized their topography and thickness of the MoS_2_ flakes using an atomic force microscope. The source/drain electrodes of the MoS_2_ FET were fabricated via e-beam lithography (EBL) using a 495 poly(methyl methacrylate) (PMMA) A2/950 PMMA A2 bilayer polymer. The MoS_2_ channel regions with dimensions 2.17 μm (length) × 3.1 μm (width) were defined using EBL. The substrate was spin-coated with 495 PMMA A2 and 950 PMMA A2 solutions, and annealed at 180 °C for 90 s after each spin coating process. Electrical contact electrodes were then established directly onto the MoS_2_ flake using EBL and substrate development. Ti/Au (5/100 nm) metal electrodes were deposited as the drain and source electrodes using an AJA hybrid magnetron sputtering system, followed by ion milling to remove the native oxide layer of the sample. A lift-off procedure was performed, resulting in the desired pattern on the substrate. Passivation was conducted using EBL to expose only the areas above the contact electrodes, enabling selective coating with polyethyleneimine (PEI) on the contact electrodes. Finally, PEI was spin-coated as a surface modifier onto the Ti/Au electrodes at 5000 rpm for 40 s, and the device was subsequently annealed at 110 °C for 10 min. The 0.5 wt% surface-modifier solution was prepared by vortexing a mixture of PEI and 2-methoxyethanol.

### Characterization

Atomic force microscopy (AFM, XE100, Park Systems), and optical microscopy (BX60M, Olympus) were used to characterize the thickness of the MoS_2_ flakes. Electrical measurements were performed under vacuum conditions (10 mTorr) using a physical properties measurement system (PPMS) in the temperature range 100–300 K. The photoelectrical properties of the MoS_2_ devices were investigated under visible-wavelength laser (405 ≤ λ ≤ 655 nm, DELOS) irradiation and under vacuum condition at room temperature.

## Results and discussion

**Fig. 1:**
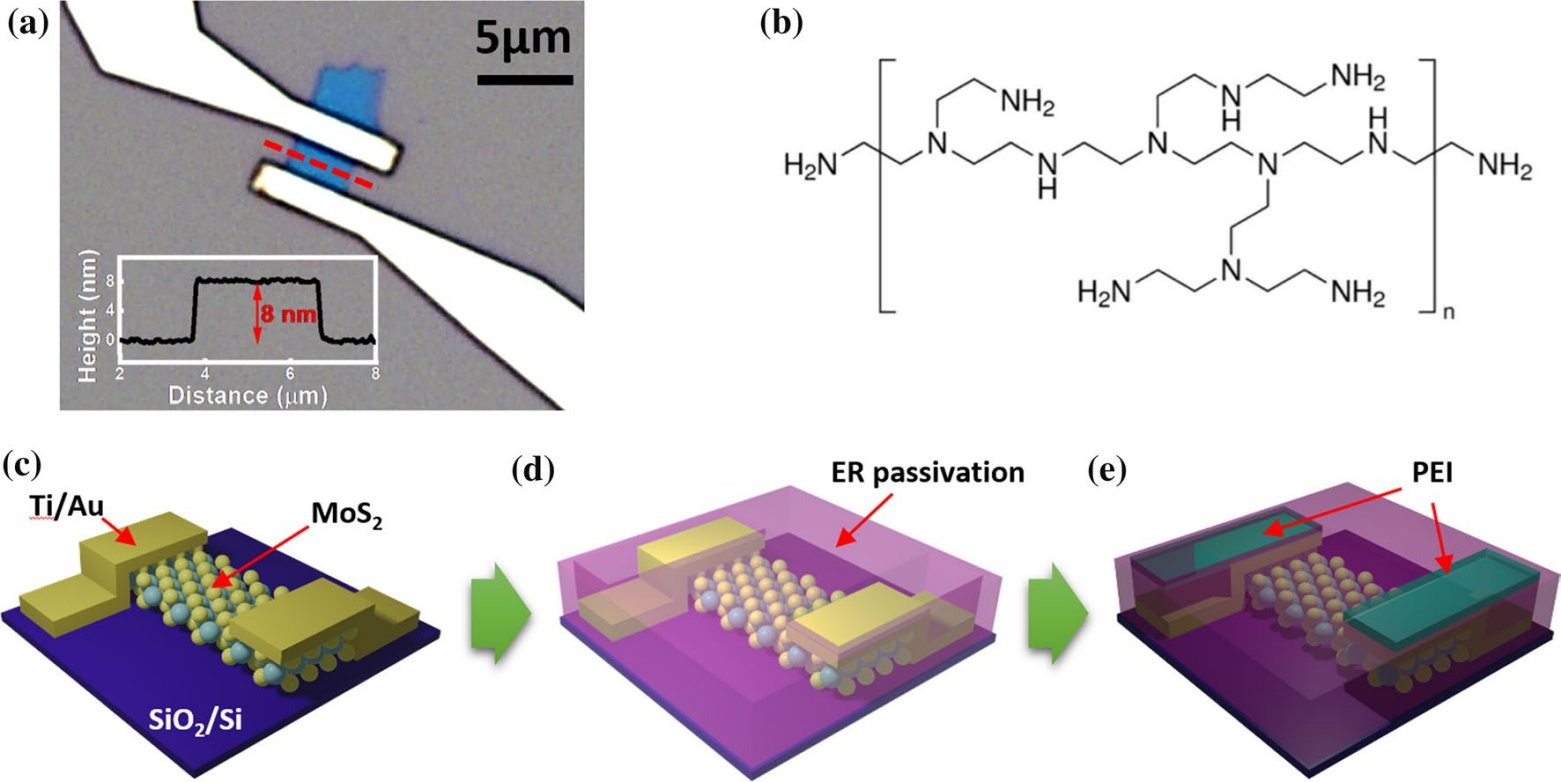
**a** Optical microscopy image of MoS_2_ FET device. The inset is AFM height profile of MoS_2_ flake. **b** Chemical structure of branched PEI used as a contact electrode modifier. **c**–**e** The schematic illustration of the contact electrode modification of the MoS_2_ device. **c** Conventional back-gated MoS_2_ FET device **d** E-beam resist passivation except for electrodes to prevent contamination of MoS_2_ channel **e** The MoS_2_ device after PEI coating on contact electrodes

Our transistor was fabricated using a top-down method known as Scotch tape-based micromechanical exfoliation of few-layer MoS_2_ flakes from bulk MoS_2_, which is a technique that facilitates the easy fabrication of high-performance devices [1, 16, 19, 34–37]. We began with bulk MoS_2_, some of which was adhered to adhesive tape, repeatedly peeled off, and transferred onto *p*-doped Si substrates covered with a 285 nm-thick SiO_2_ layer [23, 38–40]. We used an optical microscope to survey the transferred substrate to locate an MoS_2_ flake and identify the number of layers. The thickness of the MoS_2_ flake was measured using an AFM. We utilized tapping mode AFM to achieve precise thickness measurement of MoS_2_ while minimizing its damage. An optical image of the fabricated MoS_2_ FET on a Si substrate with a 285 nm-thick SiO_2_ layer is shown in Fig. 1a. Source-drain electrodes were deposited on the MoS_2_ flake to enable current flow through the MoS_2_ channel. We applied a back-gate voltage across the SiO_2_ layer to tune the Fermi level and carrier density. The inset of Fig. 1a shows the AFM height profile of an 8 nm (~ 11-layer) thick MoS_2_ flake. The channel length and width of the MoS_2_ device were 2.17 μm and 3.1 μm, respectively. To examine the characteristics of MoS_2_ FETs at various temperatures (ranging from 100 to 300 K), two-probe current-voltage (*I*-*V*) measurements were performed using a physical properties measurement system (PPMS). The electrical characteristics (transfer and output curves) of the MoS_2_ FETs were obtained using the aforementioned measurement method, and the effective barrier height and the Schottky barrier height were calculated from the results. To enable a comparison of the results before and after the application of the surface modifier, we fabricated a conventional MoS_2_ FET and then coated its surface, except for the electrode area to be treated with the surface modifier, with an e-beam resist. The electrical properties of the MoS_2_ FET were characterized conventionally and then re-measured after coating the device electrodes with PEI as a surface modifier. The PEI coating process of the MoS_2_ FET is schematically illustrated in Fig. 1c–e. The MoS_2_ FET without PEI coating is shown in Fig. 1c. The FET with an e-beam resist coating applied to prevent contamination of the MoS_2_ channel is shown in Fig. 1d. PEI coated on the Au electrodes to lower its work function is shown in Fig. 1e. Figure 1b shows the chemical structure of branched PEI used as a surface modifier. Physisorption of the surface modifier to the electrode reduced the electrode's work function. The mechanism by which work function changed is known as the interaction of the ethylamine molecular dipole (${\mu }_{\mathrm{MD}}$) and the dipole (${\mu }_{\mathrm{ID}}$) formed at the interface between the electrode surface and the molecular self-assembled monolayer (SAM). The interfacial dipole contributes to the change of the work function by promoting the transfer of some electron from the amine moieties of PEI to the electrode surface [33, 41].

**Fig. 2:**
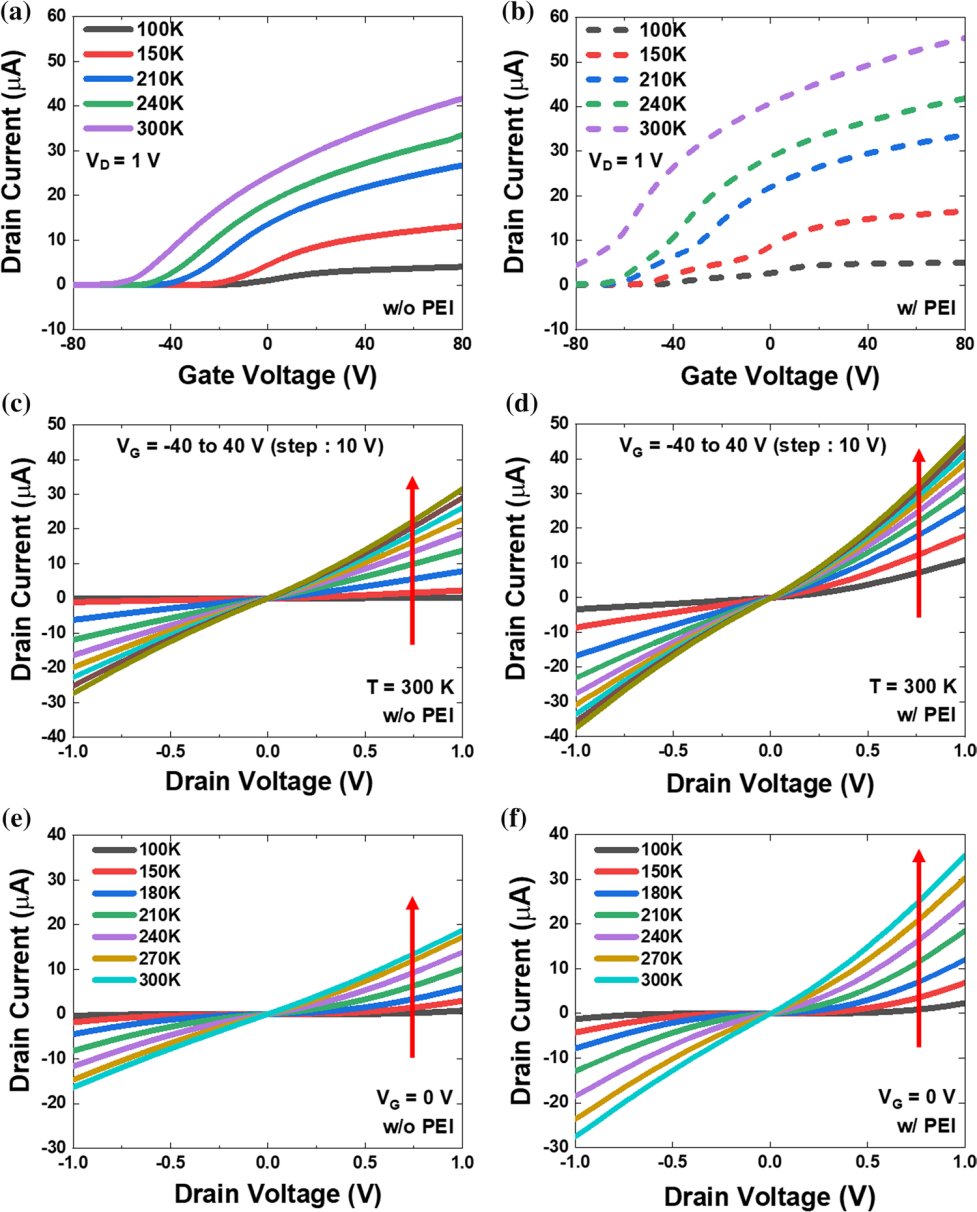
Transfer characteristics (*I*_D_-*V*_G_) of MoS_2_ FET device at *V*_D_ = 1 V **a** before and **b** after PEI coating from 100 to 300 K. Output characteristics (*I*_D_-*V*_D_) of MoS_2_ FET at 300 K **c** before and **d** after PEI coating when the gate voltage varies from − 40 to 40 V. Output characteristics (*I*_D_-*V*_D_) of MoS_2_ FET **e** before and **f** after PEI coating at *V*_G_ = 0 V from 100 to 300 K

The effect of the surface modifier on I-V characteristics was analyzed by examining the electrical characteristics of the fabricated device before and after the application of PEI. The transfer characteristics of the MoS_2_ FET are shown in Fig. 2a, b before and after the application of PEI at various temperatures. The results demonstrate *n*-type semiconductor behavior and temperature dependence. The change in the current at various temperatures can be assessed by applying a gate voltage (*V*_G_), enabling the construction of an Arrhenius plot. Both thermionic emission and tunneling contributions should be considered when evaluating the current flow through the device as a function of temperature. In general, the threshold voltage (*V*_th_) decreases as the temperature decreases. The performance of device improves as the temperature decreases, increasing *V*_th_ and decreasing junction leakage current and off-state power consumption [42]. Despite the decrese in *V*_th_ after the application of PEI, both on/off ratio and current increased. It reveals that the Schottky barrier height is reduced by processing the surface modifier, resulting in more current to flow through the device. At low *V*_G_ (*V*_th_ < *V*_FB_), the current exhibits a strong dependence on temperature, indicating that thermionic emission is the dominant mechanism. However, at high *V*_G_ (*V*_th_ > *V*_FB_), the current indicates that the field emission mechanism is dominant because of the large back-gate voltages [43].

To understand the electrical behavior of the device, we conducted two-probe drain current-drain voltage (*I*_D_-*V*_D_) measurements as a function of *V*_G_. Figure 2c, d show linear output curves (*I*_D_-*V*_D_) of the MoS_2_ FET before and after the application of the surface modifier, respectively. The results indicate that the *I*_D_-*V*_D_ output curves are strongly dependent on *V*_G_ at 300 K. At low temperatures, a non-linear output curve is observed, indicating the existence of a Schottky barrier (see Supplementary Information 1, 2). In reality, metal and MoS_2_ contacts typically have a Schottky barrier, resulting in non-ohmic behavior in the output curve. The arrow in the figures indicates the direction of increasing *V*_G_, which ranges from − 40 to 40 V in 10 V steps. The *I*-*V* curves of the MoS_2_ FET device after the application of the PEI modifier show increased linearity at low gate voltages, further indicating a greater Schottky barrier compared to the unmodified device. We also confirm that more current flows through the channel after the application of PEI than before coating at the same temperature and gate voltage. The *I*_D_ increases with increasing *V*_G_, indicating the tunability of MoS_2_ FET characteristics upon application of *V*_G_ and suggesting that the Schottky barrier is modified, which can result in the reduction of the Schottky barrier height. This interpretation agrees well with the results in Fig. 2a, b. As the temperature decreases, the output curves deviate significantly from linearity and result in a substantial decrease in *I*_D_ (see Supplementary information 3). Figure 2e–f present the output characteristics as a function of temperature at *V*_G_ = 0 V before and after the application of the surface modifier, respectively. The arrows in these figures indicate that *I*_D_ increases with increasing temperature. At the same temperature and *V*_D_, we observed a higher *I*_D_ after the application of the surface modifier. It reveals that more carriers were thermally activated and overcome the barrier to flow more current to the device after the application of the surface modifier. At high temperatures, some carriers can overcome the Schottky barrier via thermionic emission. On the contrary, at low temperatures, the drain current is proportional to the number of carriers with sufficient energy to overcome the contact barrier without thermionic emission. Therefore, the Schottky barrier height for MoS_2_ FETs can be extracted by employing the conventional thermionic emission equation,

$$J={A}^{**}{T}^{2}\mathrm{exp}\left(-\frac{q{\mathrm{\varnothing }}_{\mathrm{B}}}{{k}_{\mathrm{B}}T}\right)\left[\mathrm{exp}\left(\frac{q{V}_{\mathrm{D}}}{{k}_{\mathrm{B}}T}\right)-1\right]$$

where $$J$$ is the current density, $${A}^{**}$$ is the effective Richardson constant, *T* is the temperature, *q* is the electron charge, $${k}_{\mathrm{B}}$$ is the Boltzmann constant, *V*_D_ is the drain voltage, and *Ф*_B_ is the effective barrier height. The Richardson constant A^**^ can be expressed as $${A}^{**}=4\pi q{m}^{*}{{k}_{\mathrm{B}}}^{2}/{h}^{3}$$, where $${m}^{*}$$ is the effective mass of the MoS_2_ and ℎ is the Plank’s constant. Here, we investigate activation energy measurements that provide a way to determine the height of the Schottky barrier in a metal-semiconductor junction without assuming the size or location of the electrically active region, which can be important in investigating new or unusual interfaces. By rearranging a few terms in the above equation, we can obtain the following equation,

$$\mathit{ln}\left(\frac{{I}_{\mathrm{D}}}{{T}^{2}}\right)=\mathrm{ln}\left(A{A}^{**}\right)-\frac{q({\mathrm{\varnothing }}_{\mathrm{B}}-{V}_{\mathrm{D}})}{{k}_{\mathrm{B}}T}$$

**Fig. 3:**
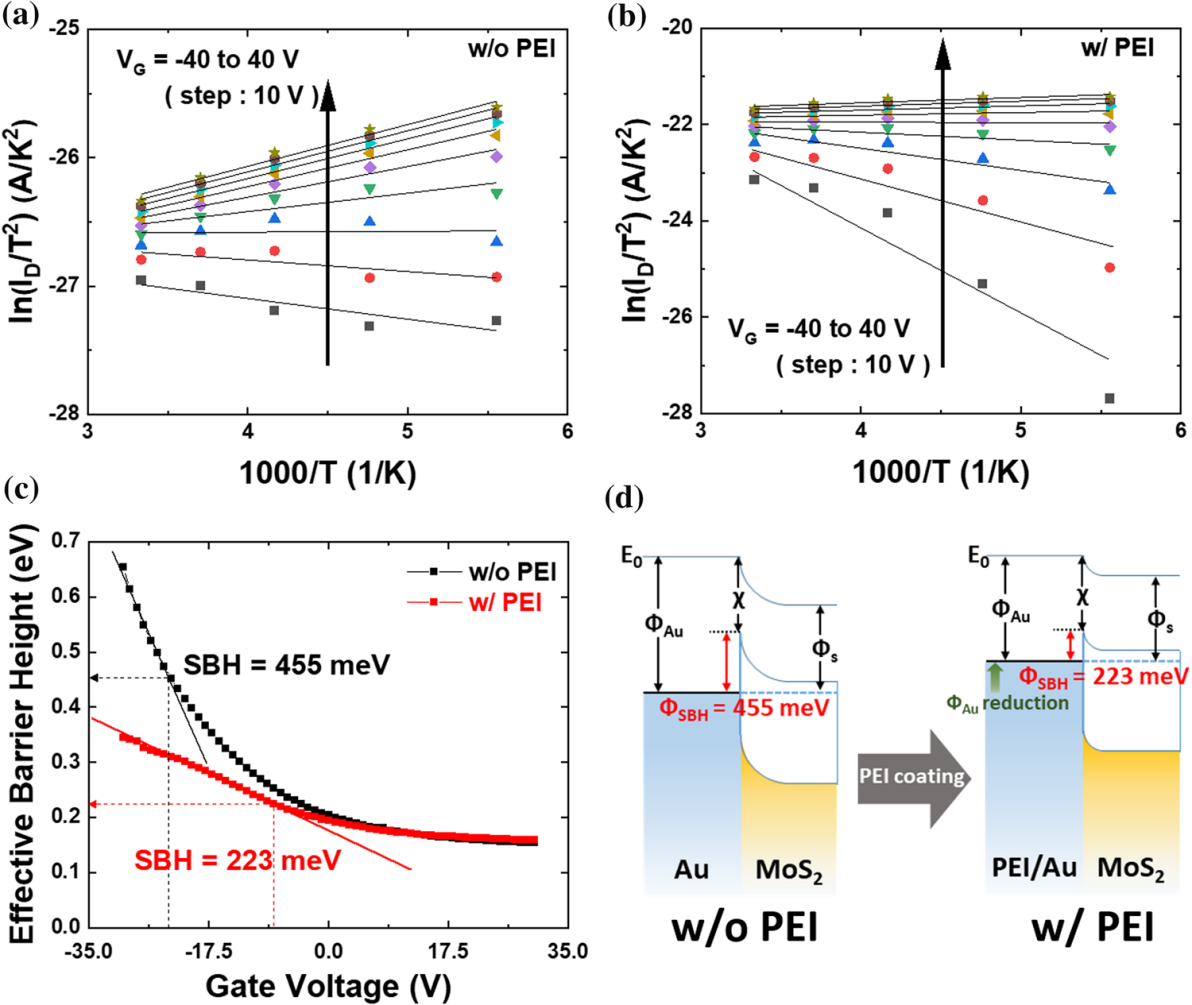
Arrhenius plots for different *V*_G_ (from − 40 to 40 V, step: 10 V) **a** before PEI coating and **b** after PEI coating. From these graphs, gate-voltage dependent barrier heights of the MoS_2_ FET is extracted. Effective barrier heights before and after PEI coating, as a function of gate voltage, obtained from **a**, **b**. **c** SBH extraction for without and with PEI coating from effective barrier heights versus *V*_G_. **d** Schematic energy band structure of the interface between contacted metal and MoS_2_ to easily show SBH reduction: before (left) and after (right) PEI coating

We constructed Arrhenius plots of $\mathrm{ln}({I}_{\mathrm{D}}/{T}^{2})$ as a function of 1000/*T* for MoS_2_ FETs before and after the application of the surface modifier (Fig. 3a, b). The data is not entirely linear across the entire temperature range. Specifically, at low temperatures, the resistance of the channel is so high that the current is not affected by the temperature, rendering the thermionic emission equation irrelevant. Therefore, the Schottky barrier height can only be extracted from the high temperature regime. The effective barrier height was extracted from the linear fitting results obtained from Fig. 3a, b (excluding 100–150 K) and plotted as a function of *V*_G_ (step: 1 V) for the device before and after the application of the surface modifier (Fig. 3c). The Schottky barrier heights are determined by the effective barrier height at the flat band voltage, which is the point where the effective barrier height begins to deviate from the linear dependence of *V*_G_. Above the flat band voltage, the thermally assisted tunneling current across the Schottky barrier can no longer be neglected, resulting in a weaker dependence of the extracted effective barrier height on *V*_G_ [23, 43]. Using this approach, the Schottky barrier height before and after the application of the surface modifier were determined as 0.45 eV and 0.22 eV, respectively (Fig. 3c). This reduction of the Schottky barrier is attributed to the lower work function resulting from the interfacial dipole of the PEI surface modifier physically adsorpted on the contact electrodes [33, 41]. Thus, the simple application of PEI coating to the Au electrodes reduced the Schottky barrier height by approximately 48%, from 0.45 to 0.22 eV. This reduction of the Schottky barrier height led to the efficient transfer of electron charge from the Au electrodes to MoS_2_, thereby considerably enhancing its electrical properties. This result confirms that electrons can be more effectively injected into the MoS_2_ channel via thermally assisted tunneling or thermionic injection. We illustrated the modulation of the Schottky barrier height with the band diagram shown in Fig. 3d. The application of the PEI surface modifier reduced the work function of the metal as a result of physisorption, which in turn reduced the band bending at the metal-semiconductor contact, thereby reducing the Schottky barrier height.

We also investigated the effect of the application of PEI on the field-effect mobility. Wang et al. [27] demonstrated that the insertion of an h-BN tunneling layer at the contacts of MoS_2_ FETs can lead to a significant improvement in their field-effect mobility because the tunneling layer reduces the height of the Schottky barrier. Their findings further suggested that minimizing contact resistance can significantly enhance the field-effect mobility of FETs, emphasizing the crucial influence of contact properties on the field-effect mobility of FETs. Therefore, we expected that the mobility would have improved because the Schottky barrier height is reduced. The field-effect mobility of MoS_2_ FETs was calculated by the following equation.

$$\mu =\left[\frac{d{I}_{\mathrm{D}}}{d{V}_{\mathrm{G}}}\right]\times \left[\frac{L}{W{C}_{\mathrm{i}}{V}_{\mathrm{D}}}\right]$$

where d*I*_D_/d*V*_G_ is the slope of the conductance curve in the linear regime, *L* = 2.17 μm is the channel length, *W* = 3.1 μm is the channel width, *C*_i_ is the capacitance between the channel and the back gate per unit area ( C_i_ = ε_0_ε_r_/d; ε_r_ = 3.9; d = 285 nm), and *V*_D_ is the drain voltage. The mobilities calculated from the transfer curves were 0.49 and 8.17 cm^2^ V^−1^ s^−1^ before and after the application of the surface modifier. We confirmed that the mobility of the MoS_2_ FET was substantially improved via the Schottky barrier height modulation.

**Fig. 4:**
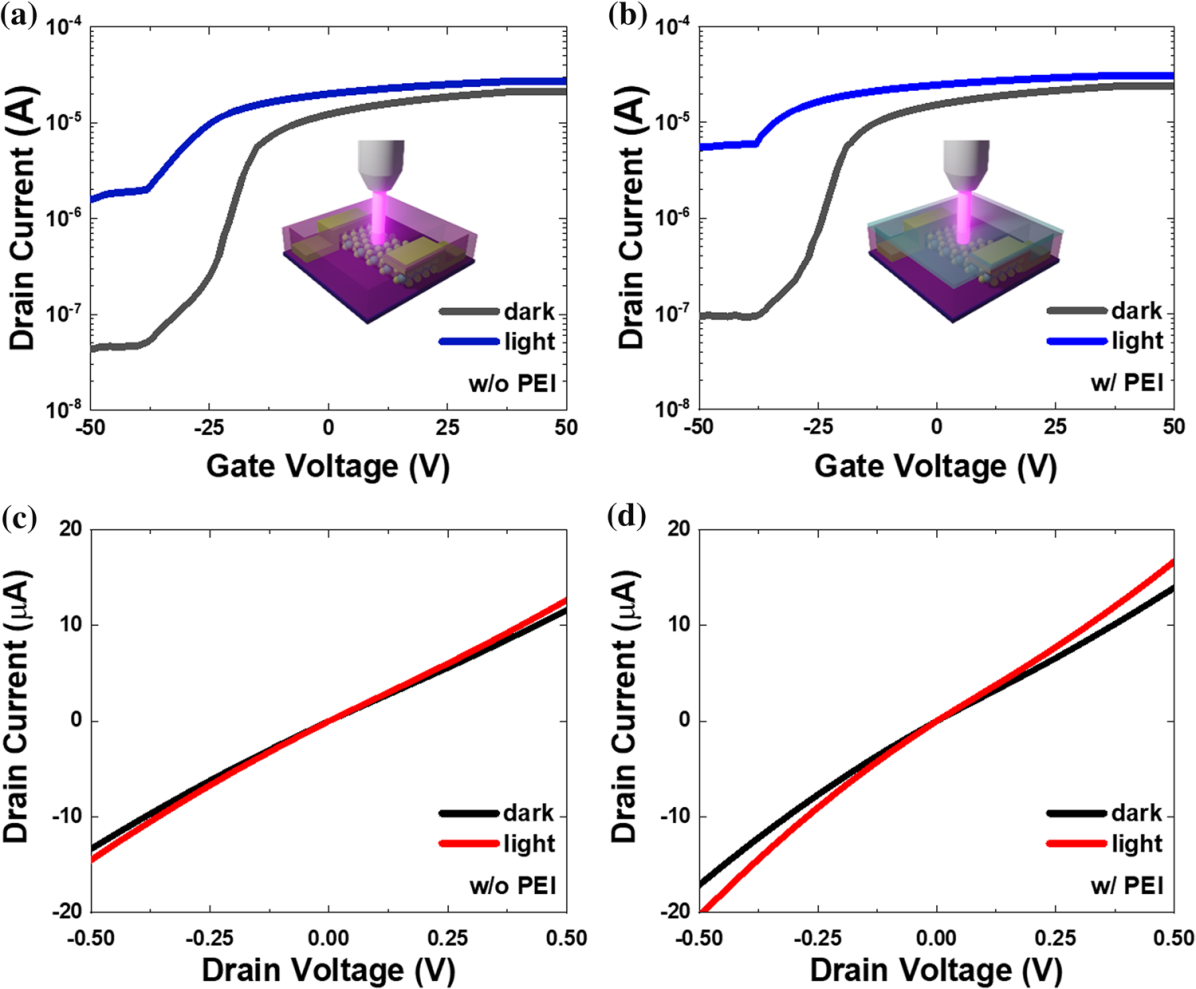
Transfer characteristics (*I*_D_-*V*_G_) of MoS_2_ photosensor **a** before and **b** after PEI coating when the laser is turned on/off. Output characteristics (*I*_D_-*V*_D_) of MoS_2_ photosensor **c** before and **d** after application of the surface modifier PEI

Finally, we demonstrated an MoS_2_ phototransistor with a reduced Schottky barrier height for the photosensor. The MoS_2_ FETs function as basic photosensors via the photoelectric effect. The photoefficiency of MoS_2_ remained unaffected as only the Au electrode was coated with PEI. This resulted in the photocurrents of both the MoS_2_ FET without PEI and with PEI being effectively increased, as depicted in Fig. 4a, b. Additionally, the current ratios of photocurrent to dark current for the devices without PEI and with PEI are comparable, indicating that the proposed PEI coating method can be applied to various channel materials used in the field of optoelectronics. When the MoS_2_ FET is irradiated with a visible-wavelength laser (405 ≤ λ ≤ 655 nm) with a greater energy level than the band gap of MoS_2_, the electrons of the electrode become excited with sufficient energy. To evaluate the photoelectric effect using only the visible-wavelength laser under dark conditions, we performed an experiment in the probe station. Figure 4a, b show the transfer curves before and after the application of the surface modifier. These figures demonstrate the difference in the on/off state of the device when the visible-wavelength laser is applied to the device before and after PEI coating. The current level increased when the laser irradiation was applied. It confirmed that our MoS_2_ device acted as a typical photosensing device. After the application of PEI, we observed that more current flowed effectively through the MoS_2_ device. Despite the generation of massive electron-hole pairs in the MoS_2_ channel under visible light, the on-current of the MoS_2_ FET increased after the application of PEI compared to before. Figure 4c, d show the results of photocurrent flowing through the channel before and after the application of PEI when the visible laser was turned on/off. They revealed that both *I*_dark_ and *I*_light_ (photocurrent) increased after the application of PEI compared to before. In addition, the photocurrent was low before PEI coating, but increased relativeldy after PEI coating as shown in Fig. 4c, d. We expect that our approach can be used for the purpose of improving the performance of photosensors by exhibiting a fast response rate while maintaining high reactivity and sensitivity at room temperature.

## Conclusions

We investigated the modulation of the effective Schottky barrier height via the surface modification of the contact electrode. We examined the reduction of the work function by applying a PEI coating and its impact on the Schottky barrier height. To compare the Schottky barrier heights before and after applying the surface modifier, we fabricated an MoS_2_ FET. The Schottky barrier height was extracted via thermionic emission analysis of the *I*-*V* curves at different temperatures (ranging from 100 to 300 K) and gate voltages (ranging from − 40 to 40 V). We found that the Schottky barrier height of the MoS_2_ FET was substantially reduced from 0.45 to 0.22 eV. This reduction arose from the physisorption of an ultrathin layer of a polymer containing simple aliphatic amine groups because of the interaction of ethylamine molecular dipole (${\mu }_{MD}$) and interfacial dipole (${\mu }_{ID}$). This transformation of the modified conductors into efficient electron-selective electrodes resulted in devices with superior electrical and photoelectrical properties compared to those of the original MoS_2_ FETs. Moreover, we found that this polymer surface modifier is an attractive alternative to incorporating chemically reactive low work function metals due to its low cost, large-area scalability, compatibility with environmentally benign solvents, and easy manufacturing process. Thus, our approach has the potential to pave the way for various electrical, photoelectrical, and organic-based electronic technologies.

## Supplementary Information

Additional file1

## Data Availability

All data generated or analyzed during this study are included in this article [and its supplementary information file].

## Abbreviations

TMDCs: Transition-metal dichalcogenides
MoS_2_: Molybdenum disulfide
FETs: Field-effect transistors
PEI: Polyethylenimine
h-BN: Hexagonal boron nitride
OSC: Organic solar cell
ITO: Indium tin oxide
PCE: Power conversion efficiency
FF: Fill factor
EBL: E-beam lithography
PMMA: Poly (methyl methacrylate)
PPMS: Physical properties measurement system
I-V: Current-voltage
SAM: Self-assembled monolayer
SBH: Schottky barrier height
