# Supplementary material for: Schottky barrier height engineering on MoS2 field-effect transistors using a polymer surface modifier on a contact electrode
Source: Discov Nano. 2023 May 31;18(1):80. doi: 10.1186/s11671-023-03855-z (PMC10232713; doi:10.1186/s11671-023-03855-z)
Supplement: Supplementary file 1 — Additional file1 [file 11671_2023_3855_MOESM1_ESM.pdf]

Supplementary Information

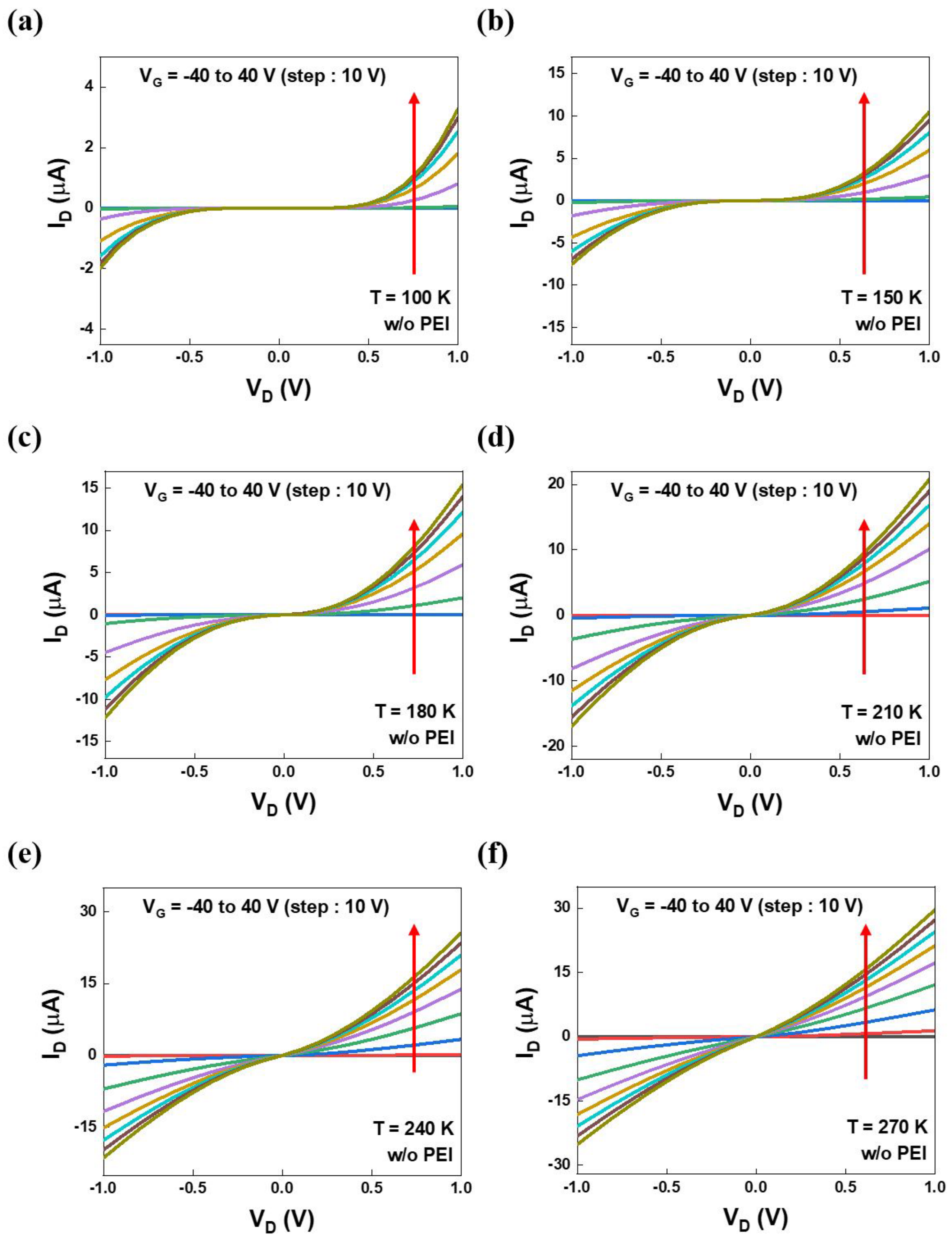

Figure 1. Output characteristics (I_D - V_D) of MoS_2 FET **(a)** at 100 K**, (b)** at 150 K**, (c)** at 180 K**, (d)** at 210 K**, (e)** at 240 K and **(f)** at 270 K before PEI coating when the gate voltage varies from -40 to 40 V.

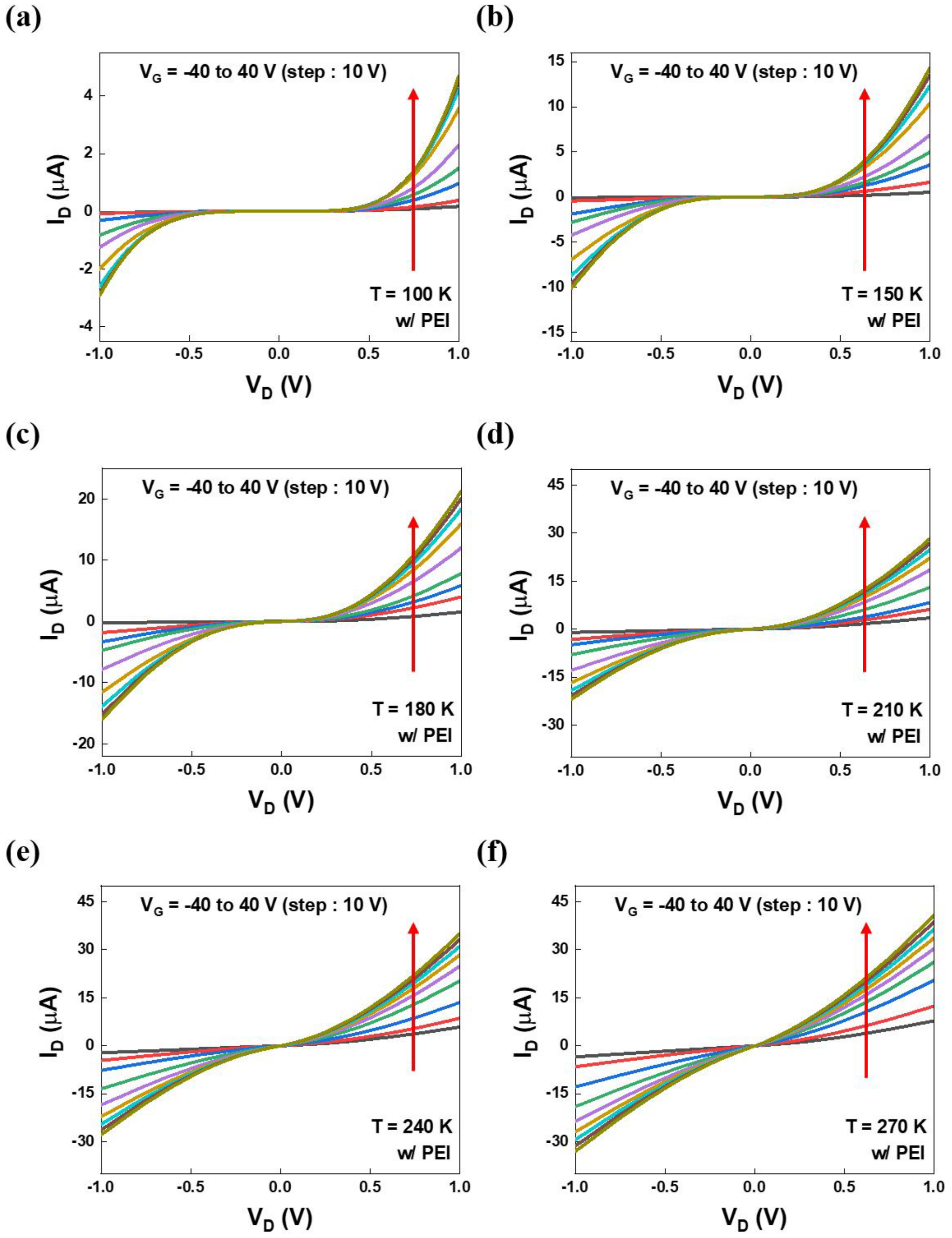

Figure 2. Output characteristics (I_D - V_D) of MoS_2 FET **(a)** at 100 K**, (b)** at 150 K**, (c)** at 180 K**, (d)** at 210 K**, (e)** at 240 K and **(f)** at 270 K after PEI coating when the gate voltage varies from -40 V to 40 V.

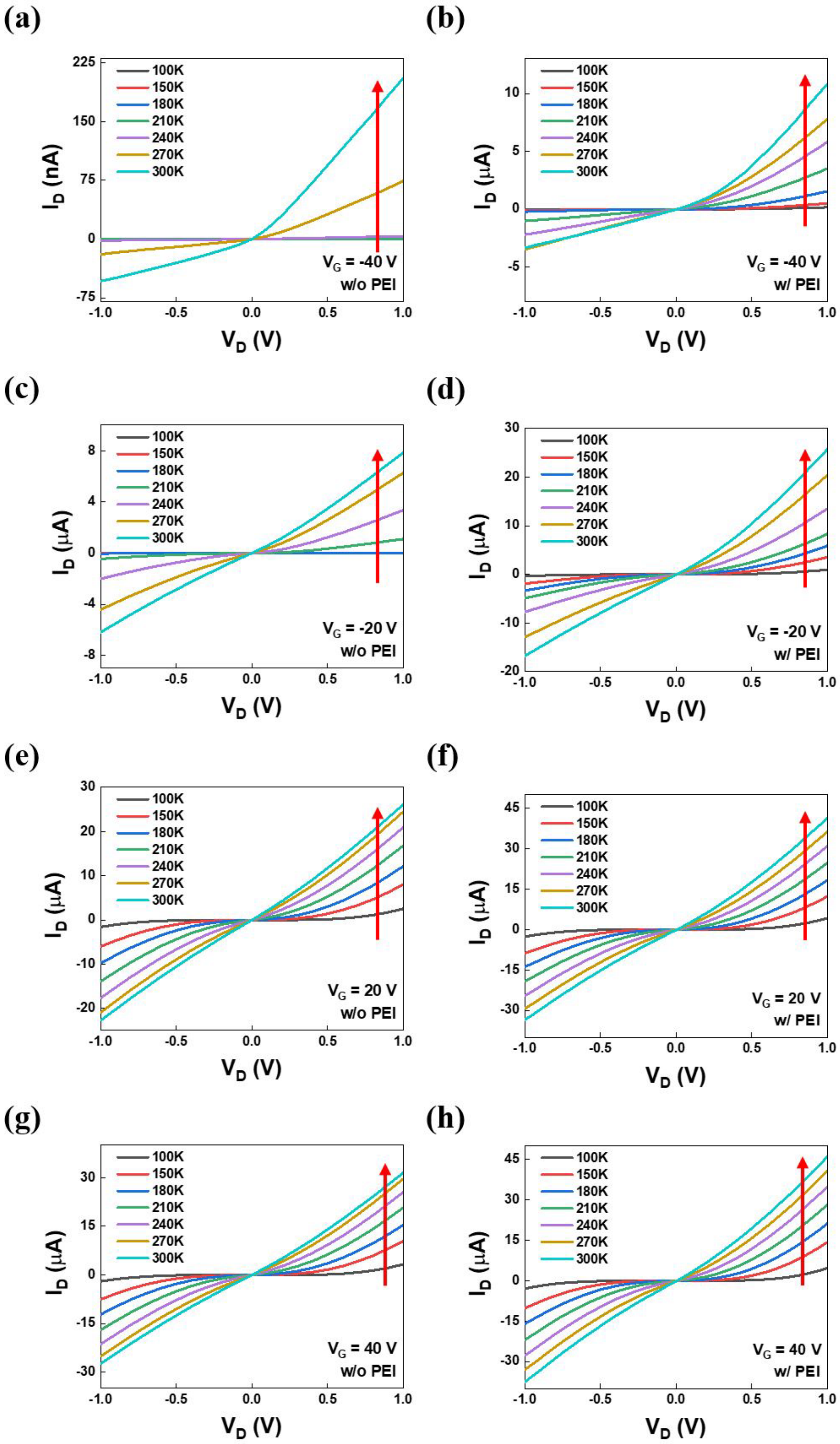

Figure 3. Output characteristics (I_D - V_D) of MoS_2 FET (**(a), (c), (e), (g)**) before and (**(b), (d), (f), (h)**) after PEI coating from 100 K to 300 K. **(a)-(b)** At V_G = -40 V, **(c)-(d)** at V_G = -20 V, **(e)-(f)** at V_G = 20 V and **(g)-(h)** at V_G = 40 V, respectively.
